# Non‐Volant Mammalian Diversity, Occurrence, and Ecological Patterns in a Tropical Montane Forest in Sarawak, Borneo

**DOI:** 10.1002/ece3.71915

**Published:** 2025-08-12

**Authors:** Mufeng Voon, Ai Suzuki, Shinya Numata, Takafumi Mizuno, Melvin Gumal

**Affiliations:** ^1^ Sarawak Forestry Corporation Kuching Sarawak Malaysia; ^2^ Graduate School of Urban Environmental Science Tokyo Metropolitan University Hachioji Tokyo Japan; ^3^ Open Innovation & Collaboration Research Organisation Ritsumeikan University Osaka Ibaraki Japan; ^4^ Graduate School of Global Environmental Studies Kyoto University Kyoto Japan; ^5^ School of Engineering and Science Swinburne University of Technology, Sarawak Campus Kuching Sarawak Malaysia

**Keywords:** activity pattern, Borneo, camera trap, high‐altitude, non‐volant mammalian, spatial partitioning

## Abstract

Mammalian species are key in maintaining a healthy ecosystem. The tropical rainforest in Borneo is characterized by its rich biodiversity and rugged interior, which houses various forest types from the lowland dipterocarp forest to the montane and ericaceous forests above 1500 m. Using the data obtained from 81 camera trap stations set up from April 2023 to September 2024, we investigated the diversity of mammalian species across the spatial and temporal dimensions. We detected 35 species of mammals from 6 orders and 15 families, excluding the Muridae and Sciuridae species. We highlight significant differences between mean species richness among tropic guilds across elevational classes. From the results of Bayesian single‐season occupancy analysis, the pig‐tailed macaque *Macaca nemestrina* has the highest occupancy rate of 0.79 (95% credible interval [CrI] 0.68, 0.89), followed by the red muntjac *Muntiacus muntjak* , 0.71 (95% CrI 0.59, 0.83). Temporally, all the individual species' activity patterns followed the previous studies, except for the mousedeer *Tragulus* spp., which are found to be mostly nocturnal. We also report evidence of differences in elevational distribution among some species within the community. In conclusion, our results offer baseline knowledge on the spatial and temporal distribution pattern of non‐volant mammals in a high‐altitude protected area.

## Graphical abstract

We investigated the diversity and activity patterns of mammals in a high‐altitude protected area in Borneo using data from 81 camera trap stations set up between April 2023 and September 2024. A total of 35 species were detected, with notable findings including a difference in mean species richness between trophic guilds, nocturnal behavior in mousedeer, and spatial partitioning between two muntjac species. These results provide important baseline knowledge on the spatial and temporal dynamics of non‐volant mammals in this rugged tropical landscape.

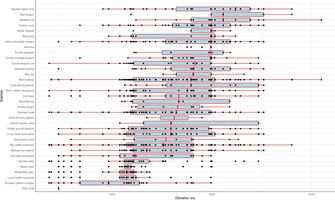

## 1 Introduction

Mammalian species play an important role in the ecosystem (Ripple et al. 2016), with carnivores regulating the population of prey species in their food chain (Ewer 1998). Herbivores, which are grazers and browsers, are often seen as “nature's gardeners.” These herbivores are involved in nutrient recycling (Pastor et al. 2006), whereas omnivores can regenerate nutrients obtained from various sources and create new nutrient pathways (Iwai et al. 2012). Every species in the ecosystem plays an important role, and these collective roles help support the balance and resilience of the ecosystem (Renison et al. 2023).

Among the world's ecosystems, mountainous regions, particularly in the tropics, are important for studying ecological and biogeographic patterns of species diversity in spatial ecology due to the rich aggregation of species (Li et al. 2021). In spatial ecology, one fundamental area of research involves understanding how species abundance and distribution respond to environmental gradients and ecological interactions (Brown 1988). In particular, elevational diversity gradients have gained more attention as an approach to examine species diversity and turnover across varying scales of biotic and abiotic conditions (Rahbek, Borregaard, Antonelli, et al. 2019; McCain and Grytnes 2010).

High‐altitude areas are usually avoided by researchers due to the costs of labor and the associated encumbrances in arranging for scarce field assistants and logistics. Furthermore, sometimes the rugged terrain can also be dangerous for field teams (Steinmetz et al. 2008). These limitations, which sometimes restrict researchers, explain why most high‐altitude forests have remained underdeveloped and not converted to other forms of land use. The high costs needed to operate in such rugged terrain often present a significant barrier (Renison et al. 2023). However, these mountainous regions are unique and high in spatial heterogeneity; therefore, they cannot be assessed using models derived from global patterns of diversity. These models are more general and cannot address the spatial variability in ecological and environmental variables, which may differ from mountain to mountain (Rahbek, Borregaard, Colwell, et al. 2019). Furthermore, the distinctive and intricate climatic conditions of these harsh mountain regions differ from those in the lowlands, likely playing an important role in maintaining the rich biodiversity of these mountainous slopes. Consequently, understanding elevational patterns is not only central to ecology and biogeography but also to conservation planning, particularly in biodiversity‐rich yet threatened tropical regions like Borneo.

The tropical rainforest in Borneo has long been known as a biodiversity‐rich hotspot, hosting a remarkable array of mammalian species, including those that are endemic and endangered (De Bruyn et al. 2014). The rugged interior area of Borneo is often characterized by steep elevational gradients and a combination of forest types, ranging from lowland dipterocarp to upper montane and ericaceous forests. Although many research activities have focused on the cryptic and endangered species of carnivores, such as Sunda clouded leopards *Neofelis diardi* , marbled cats *Pardofelis marmorata* , and bay cats *Catopuma badia* (Allen and Allan 2024; Hearn et al. 2018, 2013; Ross et al. 2013), which generally involved lowland forests, there have not been many types of research conducted on mammalian diversity and their ecology in remote montane forests. Most research has focused on high‐profile mountainous areas such as Mount Kinabalu (Nor 2001). Even so, most of these studies concentrated on systematics and taxonomy rather than ecological information such as biodiversity and ecological processes.

Besides the biogeographic aspect, it is also important to assess the temporal patterns of the species found in the mountain regions. The diel activity pattern of an animal reflects its interspecific, intraspecific, and community‐level interactions, adaptations, and habitat requirements/preferences (Vallejo‐Vargas et al. 2022). Species with overlapping resource requirements need to adapt different strategies to coexist. The mechanisms of coexistence have been studied extensively and are primarily attributed to the partitioning of resources across both spatial and temporal dimensions (Bashir et al. 2023; Sovie et al. 2019; Sunarto et al. 2015). These strategies for partitioning can be more challenging in a high‐altitude environment, challenged by the rapidly changing climate characteristic across steep elevational gradients. Ideally, understanding the organization of species communities across space and time is fundamental to the conservation of some rare and threatened species, which are sensitive to changes in environmental conditions.

This paper aims to provide insights into the species diversity of mammalian species and assess the spatio‐temporal patterns of these species in a high‐altitude montane protected forest in the Miri region of Sarawak, Borneo. It is hoped that the baseline information outlined in this paper can inform future conservation prioritization, potentially underscoring the value of elevational approaches for understanding and conserving mammalian assemblages in mountainous regions.

## 2 Materials and Methods

### 2.1 Study Area

**FIGURE 1:**
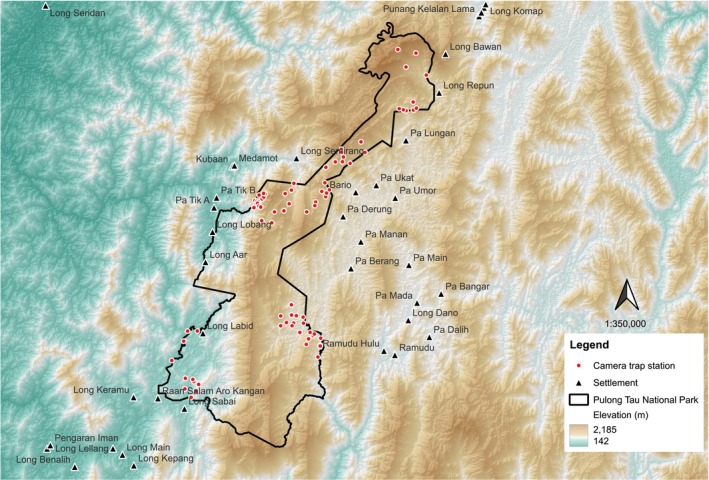
Locality map of Pulong Tau National Park and its surrounding areas.

Pulong Tau National Park (PTNP) is a totally protected area (TPA) with an area of about 598 km^2^, located in the northeastern part of Sarawak, Borneo, in the Miri division. It is situated between latitudes 3°25′26.04″ to 3°57′24.01″ and longitudes 115°12′41.87″ to 115°33′31.87″ (Figure 1). The TPA is currently managed by the Sarawak Forestry Corporation (SFC) under the National Parks and Nature Reserves Ordinance 1998. More than 63% of the area within the study is above 1200 m above sea level, with forested areas consisting of Upper Dipterocarp Forest, Montane Forest, and Ericaceous Forest (Lim 2006). Forests with elevation between 300 and 750 m are commonly known as Hill Dipterocarp Forest, whereas 750–1200 m is Upper Dipterocarp Forest (FDPM 2025). As the name suggests, the vegetation is dominated by predominantly Dipterocarpaceae. Montane Forest refers to forests from 1200 to 1500 m, and is usually dominated by *Lithocarpus* sp., *Fagaceae* sp., and *Cinnamomum* sp. Forests above 1500 m are categorized as Ericaceous Forest, dominated by species from the family Ericaceae, in addition to moss and ferns.

Based on the 24‐h daily mean temperature and daily rainfall data obtained from myMETdata, Meteorological Department of Malaysia, for the year of 2023, the mean temperature in the study area ranges from 24°C to 29°C throughout the year, whereas the average monthly rainfall is about 434.5 mm per month. Analysis of rainfall by Vijith and Dodge‐Wan (2020) reported low variation in precipitation in the Bario region.

There are 28 local communities living adjacent to the protected area, and these are mainly from three indigenous groups: the Kelabits, Lun Bawangs, and Penans. These communities rely heavily on the forest, where they hunt and gather forest produce for food, construction, and medicines. Cattle rearing and agricultural activities (growing Bario wet paddy and pineapples) are also sources of income for some within these communities.

### 2.2 Camera Trapping

Camera trapping was conducted from April 2023 to September 2024, with 81 camera trapping stations established between altitudes of 600 and 2049 m. Each station operated for an average of 99 trap nights. The camera trap stations have an average spacing of between 500 and 3000 m, calculated using the “v.distance” tool from the GRASS processing toolbox in QGIS software.

Camera traps were placed on animal trails, ridges, mountain saddles, riverbanks, and other features to maximize the detection of wildlife (Hearn et al. 2016; Lynam et al. 2013). No lures were used. The camera traps were mounted on a tree about 35–40 cm above the ground, perpendicular to the target trails, and we cleared the undergrowth as little as possible to reduce camera‐shyness behaviors in the targeted wildlife.

Camera trap models used in this survey were Browning Spec Ops Elite HP5 (75 stations), Bushnell Core S‐4K No Glow (5 stations), and Alpha Cam Dual Lens 4K No Glow (1 station). The camera traps used either Panasonic Evolta lithium‐ion batteries or Energizer lithium‐ion batteries and were set to operate on a 24‐h setting. All the Browning camera traps were configured to capture photos, whereas the Bushnell and Alpha Cam camera traps were configured to hybrid mode, capturing both photos and 10‐s videos. All the images and videos were reviewed by the team. The images were then classified into individual species folders and audited for errors. Images with ambiguous species detected were sent to various experts for confirmation and excluded from the analysis if the experts failed to identify the species. Greater and lesser mousedeer were combined as mousedeer spp. due to the difficulty in separating the species with black and white images, as the two species were mostly detected at night. Squirrels, shrews, and rats were not included in this analysis, as reliable species‐level identification was hindered by morphological similarities and a lack of specialized taxonomic expertise. The naming of all the identified species followed the nomenclature in the “Phillips Field Guide to the Mammals of Borneo and Their Ecology” (Phillips and Phillips 2016), except for the Sunda leopard cat, which follows the IUCN nomenclature due to the recent division from the mainland leopard cat *Prionailurus bengalensis* . The IUCN status of all the species is referred to from the IUCN Red List website at https://www.iucnredlist.org/.

### 2.3 Species Diversity

To ensure accuracy, we only included non‐volant mammals (excluding the Muridae and Sciuridae species) when generating the species accumulation curve. Species that displayed both ground and arboreal behavior, such as the primates, were included in the analysis. The independent events for each species per site were compiled into a matrix and analyzed using the package “vegan” in R (Oksanen et al. 2025). We used the *specaccum* function from the vegan package in R, applying randomization with 100 permutations to generate the species accumulation curve by averaging species richness across randomized sample orders.

### 2.4 Elevational Diversity Gradient

For the assessment of elevational diversity gradient, we extracted elevation data for each of the 81 camera trap stations using their geographic coordinates (WGS1984 datum) and the SRTM 1 Arc‐second Digital Terrain Elevation dataset, accessed via the USGS Global Visualization Viewer (GLOVIS). Elevation values were obtained using the “Point Sampling Tool” plugin in QGIS Desktop version 3.34.8. The stations, which ranged in elevation from 600 to 2049 m, were then grouped into fourteen 100‐m elevation class (e.g., 600–699, 700–799, …, 2000–2099 m) for subsequent analysis, with the number of stations per elevation class ranging from one to 13 stations.

To examine how species richness varies among the different trophic guilds across all elevation classes, we categorized all 34 recorded mammal species (greater and lesser mousedeer were pooled into mousedeer spp.) into one of the three guilds: carnivores, herbivores, or omnivores. The classification was based on the dietary description from the Phillips Field Guide to the Mammals of Borneo and Their Ecology (Phillips and Phillips 2016). For each of the elevation classes, we recorded the number of species detected during the survey. Within each elevation class, we calculated the mean species richness values by summing every station's species count in the band and dividing by the total number of stations in that band. By using the mean species richness per station (rather than the total unique species per band), we ensure that bands with fewer camera traps are directly comparable to bands with more traps, since each station contributes equally to the average.

This resulted in a matrix of mean richness values by guild and by elevation classes. We computed summary statistics (mean, median, range and standard deviation) of mean richness across all elevation classes. We conducted the Friedman test and the Kruskal–Wallis test, respectively to test for:

- whether species richness differs significantly between trophic guilds across elevation classes‐Friedman test.
- whether species richness within each guild varied significantly across elevation classes—Kruskal–Wallis test separately for each guild.

All analyses were conducted in R, with *p* value < 0.05 considered as significant.

Additionally, we compiled species‐specific elevation data and visualized their distributions using boxplots ordered by median elevation. To statistically test for differences, we performed a Kruskal–Wallis test across all species and followed up with Dunn's post hoc pairwise tests with Holm adjustment for multiple comparisons. The resulting adjusted *p* values were used to generate a heatmap with the pheatmap package in R, with cells annotated by adjusted *p* values and an asterisk marking significant comparisons (*p* < 0.05).

### 2.5 Species Occupancy

For the assessment of how the species occupy space, we first thinned the camera trap stations to 3 km spacing between all stations for clouded leopards and 1 km spacing for other species. This approach helps ensure spatial independence by minimizing the chance of detecting the same individual at multiple stations, following recommendations that spacing should be at least one home range diameter of the target species (Sollmann 2018). We used the Bayesian single‐season occupancy function in the R package “wiqid” (Meredith and Neelon 2022), with a sampling occasion defined as a 24‐h period (Shannon et al. 2014). The species detection history matrix for each occasion was generated in the R package “camtrapr” (Niedballa 2021) for each species. On occasions where camera traps malfunctioned with no data recorded, it was listed as “NA” in the matrix. We ran the analysis using the Bayesian framework with uninformative priors and without any covariates to estimate the probability of detection (*p*) and occupancy (psi), with 50,000 iterations used after a burn‐in of 1000 iterations to ensure model convergence. Each of the camera trap stations serves as a sampling point to accurately reflect the actual space usage of a particular species (Bailey et al. 2007; Tan et al. 2018). This method allows for the estimation of the true ratio of occupied to unoccupied areas, as compared to the conventional method using fixed plots. We only ran the analysis on species that had more than 30 independent observations. The detection of the species was then visualized in QGIS software to produce detection maps.

### 2.6 Diel Pattern Analysis

From the 81 camera trap stations, 8052 trap nights were generated. All the images from this trapping period were used in the diel pattern analysis. The mean monthly rainfall is relatively similar throughout the year (Vijith and Dodge‐Wan 2020); therefore, we assume that there are no changes in the activity patterns of wildlife within and between years.

To ensure that the images are independent from one another, only the images of a particular species detected with a 1‐h difference or more from the next image at a particular station were used for the analysis, using the overlap package in R (Ridout and Linkie 2009). First, the time of capture for each independent image was converted into radian format and then fitted to a non‐parametric kernel density function using the “densityPlot” function (for individual species) and “overlapPlot” function (for comparison of overlap between two species) to plot the probability density function of a particular image captured at a particular time. The coefficient of overlap, Dhat (Δ), represents the proportion of overlap between the diel activity curves of two species. The value of coefficients ranges from 0 (representing no overlap) to 1 (representing absolute overlap). For sample sizes that are less than 50, Dhat 1 (Δ1) coefficient is more accurate, whereas for sample sizes greater than 50, Dhat 4 (Δ4) is used as the estimator of overlap (Linkie and Ridout 2011; Ridout and Linkie 2009). An overlap coefficient higher than 0.85 is classified as a high degree of overlap; between 0.7 and 0.85 is classified as a relatively high degree of overlap, whereas less than 0.35 is classified as low degrees of overlap (Lynam et al. 2013; Rasphone et al. 2020). The confidence intervals for the coefficient were estimated using the percentile intervals from bootstrapping of 10,000 samples. We then examined the proportion of activity for specific times of the day for each species: dawn (from 5 a.m. to 6:59 a.m.), day (7 a.m. to 4:59 p.m.), dusk (5 p.m. to 6:59 p.m.), and night (from 7 p.m. to 4:59 a.m.) (Hearn et al. 2013), and classified the species into either nocturnal, diurnal, crepuscular, or cathemeral (Mohd‐Azlan et al. 2022).

## 3 Results

### 3.1 General Camera Trapping Results

Thirty‐five species of mammals from 6 orders and 15 families were recorded during the camera trapping period; 10 were endemic (Table 1). Among the species recorded, the red muntjac *Muntiacus muntjak* had the highest number of independent events (*n* = 1056) and was recorded at the most stations (*n* = 74), except for the southwestern side of PTNP, which has slightly lower elevations compared to other parts of the park. The least detected species were the North Borneo gibbon *Hylobates funereus* (*n* = 2) and the otter civet *Cynogale bennettii* (*n* = 2), both with only two independent events recorded.

**TABLE 1:** List of mammal species recorded.

	Common name	Species	Order	Family	IUCN status	Endemic	No. of independent events
1	Sunda clouded leopard	*Neofelis diardi*	Carnivora	Felidae	VU	√	33
2	Marbled cat	*Pardofelis marmorata*	Carnivora	Felidae	NT		48
3	Bay cat	*Catopuma badia*	Carnivora	Felidae	EN	√	5
4	Leopard cat	*Prionailurus javanensis*	Carnivora	Felidae			48
5	Sun bear	*Helarctos malayanus*	Carnivora	Ursidae	VU		94
6	Bearded pig	*Sus barbatus*	Artiodactyla	Suidae	VU		13
7	Red muntjac	*Muntiacus muntjak*	Artiodactyla	Cervidae	LC		1056
8	Bornean yellow muntjac	*Muntiacus atherodes*	Artiodactyla	Cervidae	NT	√	179
9	Sambar deer	*Rusa unicolor*	Artiodactyla	Cervidae	VU		126
10	Greater mousedeer	*Tragulus napu*	Artiodactyla	Tragulidae	LC		343
11	Lesser mousedeer	*Tragulus kanchil*	Artiodactyla	Tragulidae	LC		
12	Long‐tailed macaque	*Macaca fascicularis*	Primates	Cercopithecidae	EN		70
13	Pig‐tailed macaque	*Macaca nemestrina*	Primates	Cercopithecidae	VU		614
14	Red langur	*Presbytis rubicunda*	Primates	Cercopithecidae	LC	√	5
15	Hose's langur	*Presbytis hosei*	Primates	Cercopithecidae	VU	√	10
16	North Borneo gibbon	*Hylobates funereus*	Primates	Hylobatidae	EN	√	2
17	Sunda pangolin	*Manis javanica*	Pholidota	Manidae	CR		8
18	Tufted ground squirrel	*Rheithrosciurus macrotis*	Rodentia	Sciuridae	VU	√	87
19	Malayan porcupine	*Hystrix brachyura*	Rodentia	Hystricidae	LC		251
20	Bornean porcupine	*Hystrix crassispinis*	Rodentia	Hystricidae	LC	√	120
21	Long‐tailed porcupine	*Trichys fasciculata*	Rodentia	Hystricidae	LC		225
22	Yellow‐throated marten	*Martes flavigula*	Carnivora	Mustelidae	LC		203
23	Malay weasel	*Mustela nudipes*	Carnivora	Mustelidae	LC		7
24	Small‐clawed otter	*Aonyx cinereus*	Carnivora	Mustelidae	VU		5
25	Otter civet	*Cynogale bennettii*	Carnivora	Viverridae	EN		2
26	Banded civet	*Hemigalus derbyanus*	Carnivora	Viverridae	NT		269
27	Hose's civet	*Diplogale hosei*	Carnivora	Viverridae	VU	√	165
28	Malay civet	*Viverra tangalunga*	Carnivora	Viverridae	LC		96
29	Island palm civet	*Paradoxurus hermaphroditus*	Carnivora	Viverridae	LC		12
30	Masked palm civet	*Paguma larvata*	Carnivora	Viverridae	LC		70
31	Banded linsang	*Prionodon linsang*	Carnivora	Prionodontidae	LC		44
32	Binturong	*Arctictis binturong*	Carnivora	Viverridae	VU		18
33	Moonrat	*Echinosorex gymnurus*	Eulipotyphla	Erinaceidae	LC		60
34	Short‐tailed mongoose	*Herpestes brachyurus*	Carnivora	Herpestidae	NT		112
35	Collared mongoose	*Herpestes semitorquatus*	Carnivora	Herpestidae	NT	√	34

**FIGURE 2:**
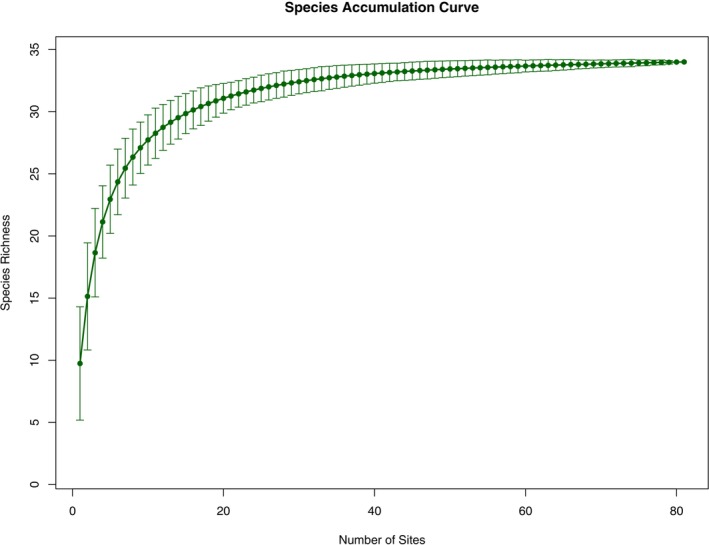
Species accumulation curve for Pulong Tau National Park. Bars indicate standard deviations.

Among the four felid species recorded, the leopard cat *Prionailurus javanensis* and the marbled cat *P. marmorata* both shared the highest number of independent events (*n* = 48). The leopard cat, however, was recorded at 24 stations, whereas the marbled cat was recorded at 15. We also detected species that are believed to be fully or partially arboreal such as red langurs *Presbytis rubicunda*, Hose's gray langurs *Presbytis hosei* , and North Borneo gibbons, despite the camera traps being set for terrestrial species. We managed to reach an asymptote on the species accumulation curve (Figure 2), indicating sufficient effort was covered in this study and estimating 35 species of mammals in the study site.

### 3.2 Elevational Diversity Gradient

**FIGURE 3:**
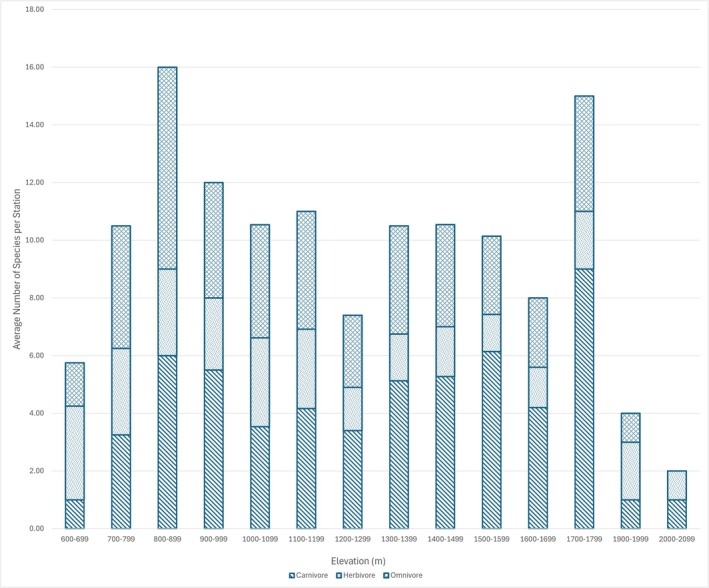
Mean species richness by trophic guild across elevation bands in Pulong Tau National Park. The plot shows changes in mean species richness along an elevation gradient for carnivores, herbivores, and omnivores.

Descriptive statistics indicated clear differences in mean richness values among the tropic guilds (Figure 3), with carnivores having the highest mean richness value of 4.19 species per elevation class (SD = 2.26); followed by omnivores with a mean richness of 3.19 (SD = 1.71), and herbivores with the lowest mean richness value of 2.15 (SD = 0.76).

The Friedman test revealed a statistically significant difference in species richness between the three trophic guilds across elevation bands (*χ* ^2^ = 9.15, *p* = 0.0103), confirming that the observed differences in richness were unlikely due to random variation. However, Kruskal–Wallis tests conducted independently for each guild showed that species richness within each guild did not vary significantly across elevation (*χ* ^2^ = 13.00, *p* = 0.448).

**FIGURE 4:**
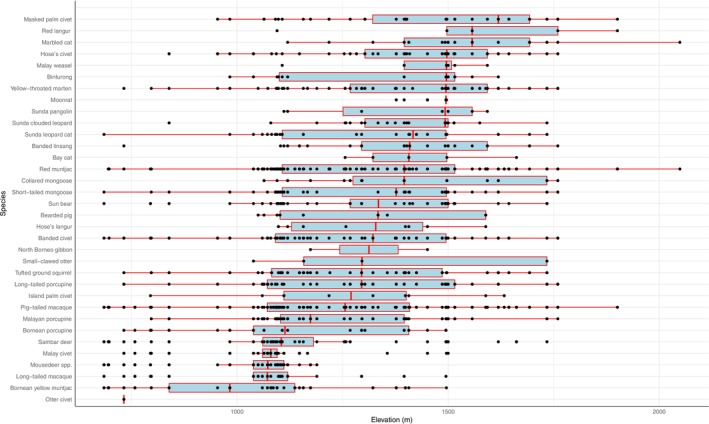
Elevation distributions of species recorded in Pulong Tau National Park. Boxplots show the median, interquartile range, and overall spread of elevations for each species, ordered from highest to lowest median elevation. Jittered points represent individual detections.

**FIGURE 5:**
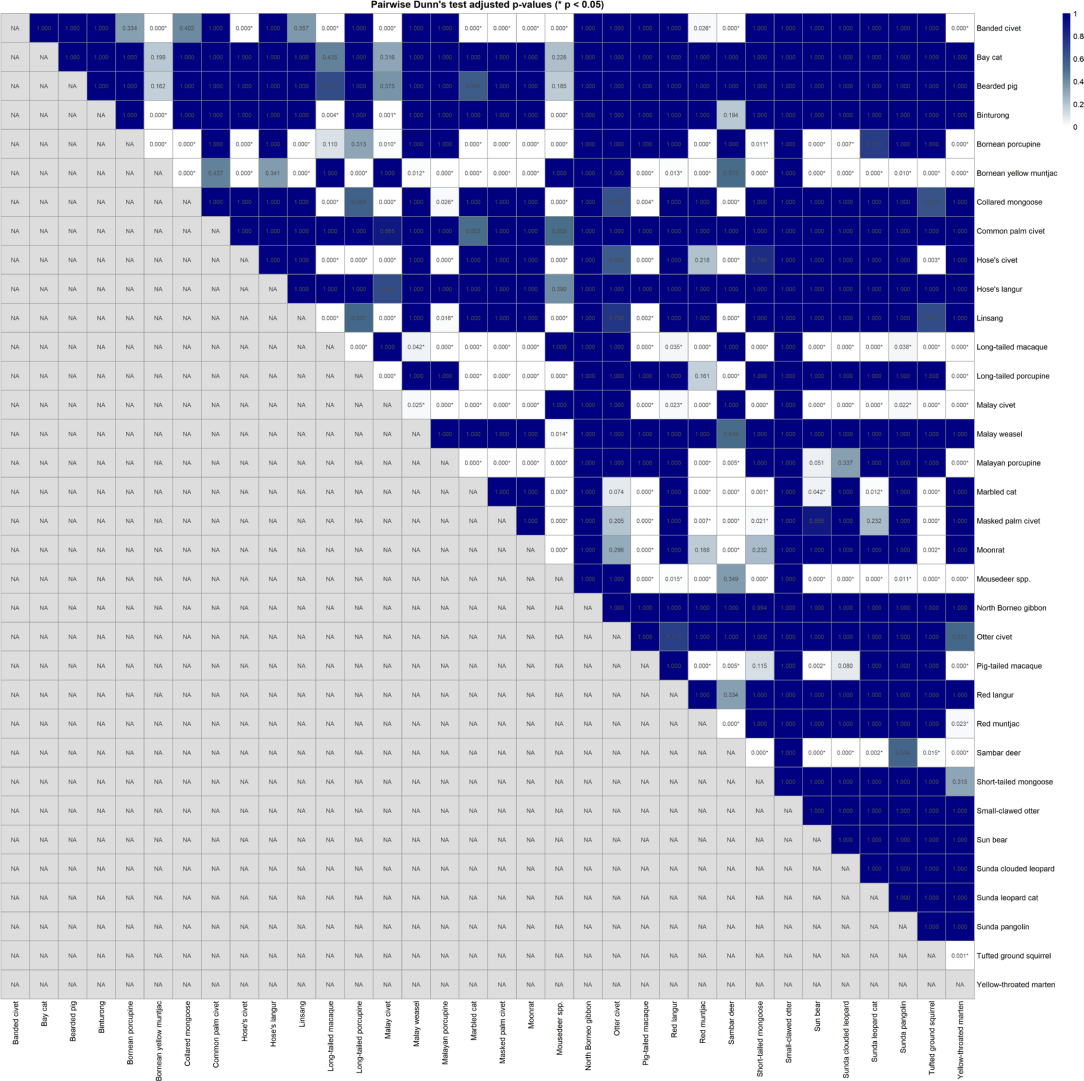
Pairwise differences in elevation distributions among species based on Dunn's post hoc tests. The heatmap shows Holm‐adjusted *p* values from pairwise Dunn's tests following a significant Kruskal–Wallis analysis (*H* = 1112.88, *p* < 2 × 10^−212^) comparing elevation distributions across species. Cells are annotated with adjusted *p* values, and an asterisk (*) indicates significant differences at adjusted *p* < 0.05.

Masked palm civets *Paguma larvata* are predominantly recorded at higher elevations than other species, followed by red langurs (Figure 4). Conversely, the Malay civets *Viverra tangalunga* , mousedeer spp., long‐tailed macaques *Macaca fascicularis* , Bornean yellow muntjacs *Muntiacus atherodes* , Bornean porcupines *Hystrix crassispinis* , and otter civets were recorded at relatively lower elevations. The Kruskal–Wallis test revealed highly significant differences in elevation distributions across species (*H* = 1112.88, *p* < 2 × 10^−212^), indicating pronounced elevational structuring within the mammalian community. Subsequent Dunn's post hoc pairwise tests, adjusted using the Holm method, identified numerous significant differences (adjusted *p* < 0.05) between species pairs (Figure 5). The complete matrix of pairwise adjusted *p* values is provided in Table S1.

### 3.3 Species Occupancy

The occupancy rate varies across different species. The most widespread species in the study site is the pig‐tailed macaque, with a mean probability of occupancy (mean *ψ*) of 79% (CrI of 67.7%, 89.4%), followed by the red muntjacs, 71.3% (CrI of 58.9%, 83.1%); see Table 2.

**TABLE 2:** The naïve occupancy and Bayesian occupancy estimate, psi with null model for the selected mammal species detected.

Species	Naïve estimate	Bayesian occupancy with null model, psi	95% CrI
Pig tailed macaque	0.7692308	0.79	0.677, 0.894
Red muntjac	0.6923077	0.713	0.589, 0.831
Leopard cat	0.2307692	0.712	0.394, 1
Yellow throated marten	0.5192308	0.587	0.439, 0.736
Banded civet	0.5	0.542	0.402, 0.683
Hose's civet	0.4230769	0.487	0.338, 0.64
Tufted ground squirrel	0.3461538	0.487	0.294, 0.691
Clouded leopard	0.1176471	0.46	0.0735, 0.945
Malayan porcupine	0.4230769	0.451	0.316, 0.588
Short tailed mongoose	0.2692308	0.427	0.217, 0.663
Sambar	0.3076923	0.414	0.24, 0.6
Banded linsang	0.2115385	0.401	0.162, 0.699
Bearded pig	0.05769231	0.397	0.0404, 0.916
Sun bear	0.2692308	0.38	0.204, 0.566
Collared mongoose	0.1153846	0.34	0.0664, 0.761
Long tailed porcupine	0.2884615	0.309	0.189, 0.437
Mousedeer	0.2692308	0.291	0.171, 0.415
Malay civet	0.25	0.285	0.162, 0.414
Long tailed macaque	0.1730769	0.279	0.109, 0.477
Binturong	0.09615385	0.278	0.0437, 0.673
Yellow muntjac	0.25	0.273	0.155, 0.395
Masked palm civet	0.2115385	0.268	0.137, 0.406
Marbled cat	0.1538462	0.215	0.0873, 0.356
Bornean porcupine	0.1153846	0.143	0.053, 0.243
Moonrat	0.03846154	0.0579	0.0639, 0.121

**FIGURE 6:**
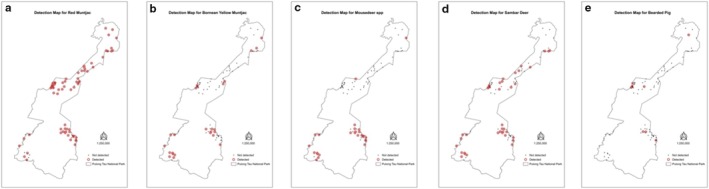
Detection map for (a) red muntjac, (b) Bornean yellow muntjac, (c) mousedeer, (d) sambar deer, and (e) bearded pig.

The detection maps were generated to visualize the detection of the species spatially. For the felids, the most widespread leopard cats were detected at 24 stations, followed by the clouded leopards *N. diardi* at 20 stations, the marbled cat at 15 stations, and the bay cat *C. badia* at five stations. The detection maps for the felid species are not included in this manuscript for the protection of these species from poaching and illegal wildlife trade. For the ungulate species, bearded pigs *Sus barbatus* were detected at eight stations, sambar deer *Rusa unicolor* at 38 stations, red muntjac at 72 stations, Bornean yellow muntjac *Muntiacus atherodes* at 25 stations, and mousedeer spp. at 26 stations (Figure 6).

### 3.4 Diel Activity Pattern

**FIGURE 7:**
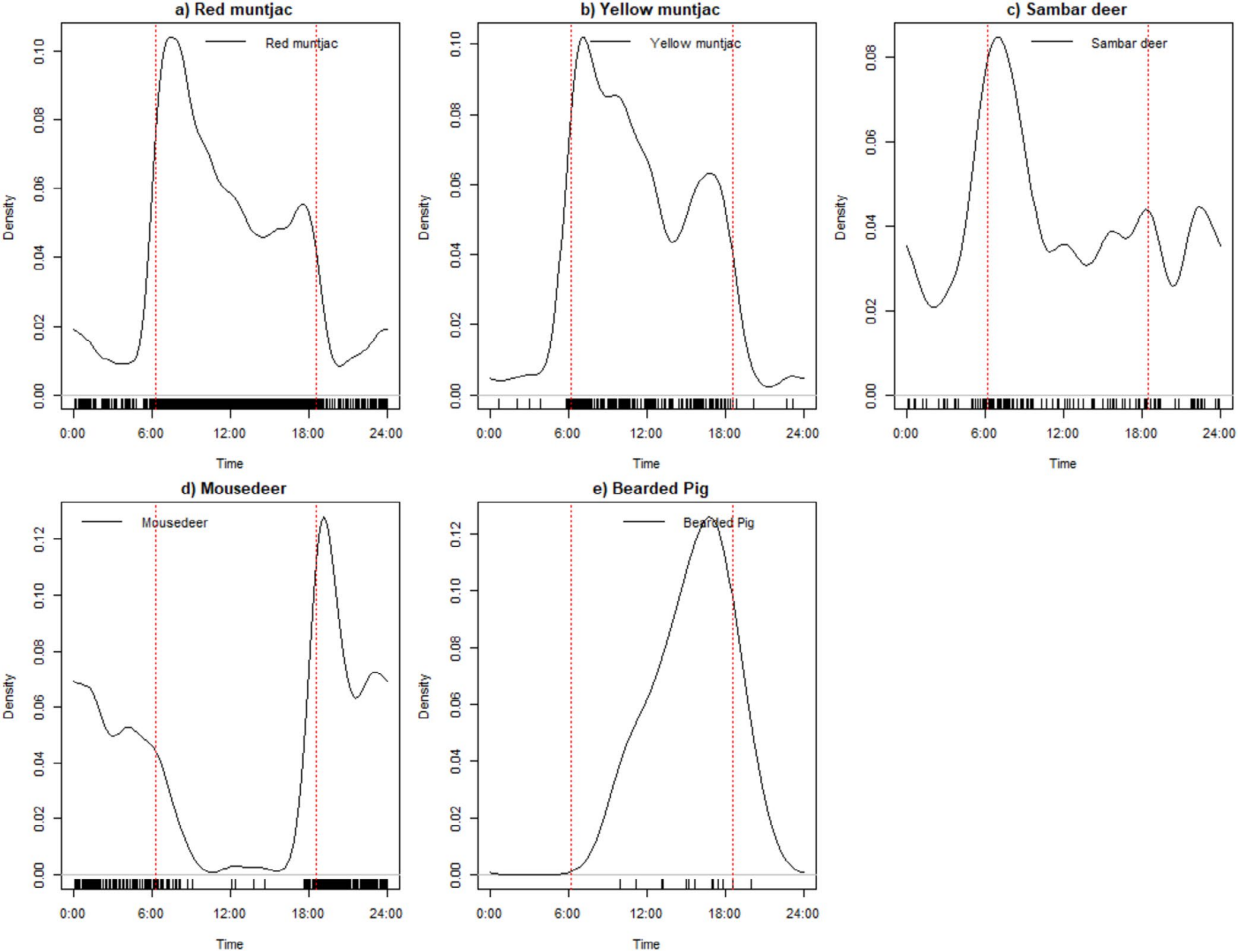
Kernel density estimates of the daily activity patterns of each of the ungulate species recorded: (a) red muntjac, (b) Bornean yellow muntjac, (c) sambar deer, (d) mousedeer spp., (e) bearded pig. The time of each independent event is indicated by short vertical black lines above the *x* axis. The average sunrise time (6.20 a.m.) and sunset time (6.33 p.m.) are indicated by two red lines on the graphs.

For felids, the leopard cats are nocturnal (22% of activities during the day), followed by the clouded leopard, which is mostly nocturnal (29% of activity recorded during the day), whereas marbled cats (79%) are diurnal. Although bay cats only have five independent records, it is worth noting that all five records were taken during the day. For ungulates (Figure 7), the yellow and red muntjacs and bearded pigs are diurnal (82.8%, 78.1%, and 81.4%, respectively). Mousedeer spp. are nocturnal (19.0% of activities recorded during the day), whereas sambar deer have 55.8% of activities recorded during the day, making them cathemeral. The pangolin *Manis javanica* , all porcupines, and civet species are strongly nocturnal, whereas Binturong *Arctictis binturong* and sun bear *Helarctos malayanus* are cathemeral. Both species of mongoose and the Malay weasel *Mustela nudipes* are strongly diurnal (Table 3).

**TABLE 3:** Diel pattern of the mammal species.

Species	Day	Night	No of independent captures	Activity pattern
Red muntjac	78.1%	21.9%	1056	Diurnal
Pig tailed macaque	93.9%	6.1%	614	Diurnal
Mousedeer	19.0%	81.0%	343	Nocturnal
Banded civet	4.0%	96.0%	269	Nocturnal
Malayan porcupine	2.3%	97.7%	251	Nocturnal
Long tailed porcupine	1.8%	98.2%	225	Nocturnal
Yellow‐throated Marten	90.3%	9.7%	203	Diurnal
Yellow muntjac	82.8%	17.2%	179	Diurnal
Hose's civet	4.1%	95.9%	165	Nocturnal
Sambar deer	55.8%	44.2%	126	Cathemeral
Bornean porcupine	3.3%	96.7%	120	Nocturnal
Short‐ tailed mongoose	92.9%	7.1%	112	Diurnal
Malay civet	21.1%	78.9%	96	Nocturnal
Sun bear	65.0%	35.0%	94	Cathemeral
Tufted ground squirrel	97.2%	2.8%	87	Diurnal
Masked palm civet	15.0%	85.0%	70	Nocturnal
Long tailed macaque	92.0%	8.0%	70	Diurnal
Moonrat	8.4%	91.6%	60	Nocturnal
Leopard cat	21.0%	79.0%	48	Nocturnal
Marbled cat	79.4%	20.6%	48	Diurnal
Linsang	2.9%	97.1%	44	Nocturnal
Collared mongoose	88.1%	11.9%	34	Diurnal
Clouded leopard	29.2%	70.8%	33	Nocturnal
Binturong	56.6%	43.4%	18	Cathemeral
Bearded pig	81.4%	18.6%	13	Diurnal
Pangolin	1.5%	98.5%	8	Nocturnal
Malay weasel	81.5%	18.5%	7	Diurnal
Bay cat	97.7%	2.3%	5	Diurnal

In terms of overlapping activities between species (focusing on overlap greater than 0.75), the clouded leopard's activities overlapped the most with the Malay civet *Viverra tangalunga* (ΔDhat1 = 0.790), followed by the leopard cat (ΔDhat1 = 0.781). The marbled cat has the highest overlap with the red muntjac (ΔDhat1 = 0.888), followed by the Bornean yellow muntjac (ΔDhat1 = 0.879), pig‐tailed macaque *Macaca nemestrina* (ΔDhat1 = 0.814), short‐tailed mongoose *Herpestes brachyurus* (ΔDhat1 = 0.806), yellow‐throated marten *Martes flavigula* (ΔDhat1 = 0.768), and long‐tailed macaque (ΔDhat1 = 0.756). The leopard cat has the most overlap with the masked palm civet (ΔDhat1 = 0.845), followed by the mousedeer (ΔDhat1 = 0.835). The overlap between both muntjac species has a high degree of overlap, with a coefficient of 0.9189 (95% CI: 0.8762, 0.9615).

## 4 Discussion

The species accumulation curve has reached an asymptote within the number of camera trapping stations of this study, suggesting that the trapping effort is sufficient to detect the non‐volant mammal species, excluding the rats, squirrels, and shrew species. A similar study conducted in tropical lowland and lower montane forests in southern Sarawak recorded 22 mammal species (Kaicheen and Mohd‐Azlan 2022); whereas research carried out on Mount Kinabalu in Sabah, Borneo, reported 12 mammal species (Nor 2001). Compared to these studies, the findings from PTNP suggest that non‐volant mammal species richness is relatively high, highlighting the park's potential as an important biodiversity refuge and hotspot within the region.

### 4.1 Elevational Diversity Gradient

Previously, researchers assumed that the lowland tropical rainforest could sustain a greater number of species and that species diversity declines with an increase in altitude, mirroring the latitudinal gradient (e.g., Mani and Mani 1968; Begon et al. 1990). The impact of elevation on wildlife is not direct. Instead, elevation is a proxy for a few elevation‐correlated environmental parameters such as historical and present geometric characteristics, precipitation, soil moisture, and rainfall, among other variables (Rahbek 1995). Environmental gradients (e.g., temperature, humidity, and vegetation structure) differ considerably with elevation (Hazebroek et al. 2004) to the extent that different environments may provide niches for a variety of mammal species. The patterns of species richness observed along the elevational gradient are likely to reflect the ecological complexity and environmental conditions endemic to PTNP, which were not investigated in this study.

Our results show that species richness varied among the trophic groups, with carnivores having the highest mean species richness value. These differences observed suggest that the mammal community structure in PTNP may be shaped by the trophic roles. The high species richness in the carnivore species may reflect the larger home ranges and broader habitat use (Noss et al. 1996). For example, many carnivores may need to track their prey across a wide range of elevations (Carbone and Gittleman 2002). In contrast, herbivores may be more limited by vegetation type and food availability, making them more restricted to a certain elevation range. They may also be more susceptible to hunting pressure compared to other species, especially in areas easily accessible by hunters (Ripple et al. 2016). Therefore, while patterns are visually and ecologically suggestive, they must be interpreted with caution, and we recommend denser, evenly distributed sampling in future studies to validate these findings. Although we observed a difference between trophic groups across the elevational gradient, there was no significant variation observed within each of the guilds across elevations. This may indicate that trophic groups maintain a relatively stable distribution across elevation, and further research is needed to investigate the reasons for this stability.

The species seem to portray a nested subset pattern of environmental requirements and ranges, where most species overlap at the center of the elevational gradient, with differences in the spread of the gradient, as suggested by Brown (2001). This observation may be because the mid‐elevations with higher precipitation may be more productive and conducive to supporting communities of species with the increase of resources (Brown 2001; Heaney 2001). Although this study is limited in spatial scale and may not be robust enough to draw any conclusions, it updates, on a local scale, the existing knowledge on the elevational diversity gradient, which has been acknowledged as an important tool to assess the patterns of species biodiversity and ecosystem functions (Guo et al. 2013; Sanders and Rahbek 2012; Nogués‐Bravo et al. 2008).

### 4.2 Spatial Pattern

In terms of occurrence, the clouded leopard (*n* = 20) has a higher probability of occupancy in PTNP than the marbled cat (*n* = 15); although the number of independent events for the marbled cat is slightly higher (*n* = 48) compared to the clouded leopard (*n* = 33). Clouded leopards were detected throughout PTNP from the northeastern part to the southwestern part of Long Sabai, potentially because they are apex predators and the overlap in resources with other felid species is manageable. The areas where clouded leopards are recorded are mostly mountain ridges with distinctive cliffs on either side of the ridge, further confirming the observation that felids prefer to travel on ridges or trails (Wearn et al. 2013). For felid species of similar body size, a certain degree of spatial partitioning is observed. Marbled cats were not detected in the southwestern part of PTNP, that is, where leopard cats were detected. The leopard cats were detected spanning a wider elevational gradient than the marbled cats. A similar observation was recorded in previous research in Indonesia, assessing the spatial and temporal interactions of these mesopredators (Haidir et al. 2018).

Although not directly investigated in this study, the results indicated some form of horizontal and vertical scale of partitioning, which may potentially be attributed to mechanisms of prey and predator relationships to cope with resource competition. Vanak et al. (2013) found that carnivore species adapt their spatial strategies and behavior based on areas with the highest probability of resource acquisition while minimizing competition with other intraguild competitors that share the same prey base. Similarly, in Indonesia, it was observed that a plausible mechanism to avoid resource competition is for smaller carnivore species to shift vertically up the elevational gradient (Sunarto et al. 2015). They observed that with the presence of a large carnivore, such as tigers, the smaller‐sized carnivore, such as clouded leopards, which may overlap in prey base, tends to shift to a higher elevation than tigers, as well as golden cats, which are found at even higher elevations than clouded leopards. Other researchers also reported similar observations, where clouded leopards are distributed at a lower elevation than other smaller felids, such as marbled cats and golden cats, which are found at relatively higher elevations (Pusparini et al. 2014). Steinmetz et al. (2008) hypothesized that the differences observed between elevation zones could also be due to variations in vegetation structure, adequacy of resources, and other anthropogenic factors, which may contribute to shaping these mechanisms.

For the non‐felid species, such as the red and yellow muntjac, although they have similar diel activity patterns, they occupy statistically different elevational ranges, suggesting a form of spatial niche partitioning. This could potentially indicate that segregation along the elevational gradient may be a strategy to reduce competition and co‐exist (Petersen et al. 2019; Vanak et al. 2013). This form of allopatric habitat use within a common landscape is a common strategy among congeneric or ecologically similar species, particularly in a rugged environment.

### 4.3 Temporal Pattern

Temporal partitioning is observed within the felid guild, such as marbled cats, which are mostly diurnal; leopard cats, which are predominantly nocturnal; while bay cats occupy intermediate or less overlapping temporal niches with marbled cats. Such behaviors were similarly observed by Mohamed et al. (2013) in Sabah, Borneo. Researchers believe this to be an important mechanism to facilitate the coexistence of felids with similar body mass (Ross et al. 2013; Sunarto et al. 2015). These species often share similar prey bases, including small mammals, birds, and rodents, which potentially lead to intense inter‐species competition (Rabinowitz et al. 1987; Sovie et al. 2019). As a mitigation to the limited resources, these felids may effectively divide their temporal patterns to prevent direct encounters and competition, thus allowing multiple sympatric species to thrive without affecting each other's foraging success (McCarthy et al. 2015).

Both species of muntjacs in PTNP are mostly diurnal, with activity peaking right after sunrise and declining during noon, followed by another small peak before sunset. These observations are similar to the research by Tan et al. (2018) in Peninsular Malaysia and Bersacola et al. (2019) for studies in Kalimantan, where a decline in activity during the afternoon was observed. This can potentially be attributed to mechanisms to prevent overheating by limiting their activity during the hottest times of the day, especially for species with higher body mass, as reported by Vallejo‐Vargas et al. (2022) when investigating the diel activity pattern of forest mammals in the tropical region.

When compared with other studies conducted in other parts of Borneo, we found some dissimilarities. For example, Bersacola et al. (2019) found that in Kalimantan, Borneo, the mousedeer has an almost equal activity pattern during the day and night. They also reported a positive correlation between the diurnality of mousedeer and muntjac with the detection rates of predators such as clouded leopards. Meanwhile, in PTNP, the mousedeer are strongly nocturnal, with activity peaks after sunset and before sunrise. Another study in Kalimantan assessed the activity patterns of mousedeer within conservation fragments of oil palm plantations, showing that the species is active throughout the day, with the highest activity peak during the daytime (Kasper et al. 2024). Based on radio‐collaring data of lesser mousedeer in Sabah collected by Matsubayashi et al. (2003), the previously thought nocturnal species was found to be most active during the day, engaging in foraging and moving between shelters, and resting at night on higher ridges. Conversely, in PTNP, the activity overlap of mousedeer and clouded leopard is as high as 72% (95% confidence interval: 59% and 85%), and both species demonstrated strong nocturnal activity patterns, with some daytime activity.

Generally, researchers reported the diel activity pattern of wildlife is determined by evolution but adjusted based on the responses to the severity of environmental stressors, that is, predation risks (Kronfeld‐Schor and Dayan 2003; Monterroso et al. 2013). Monterroso et al. (2013) reported that although the predation risk for murids is higher at night than during the day, the murids consistently displayed a nocturnal activity pattern. They suggested that being active during high‐risk periods such as nighttime may potentially benefit the survival of the overall prey population by increasing accessibility to prey. Another study in Africa found that the herbivores adapt their foraging activities to hotter times of the day when exposed to predation risk during the night, suggesting trade‐offs between thermal constraints and predation risk (Veldhuis et al. 2020), consistent with research from Tambling et al. (2015) who also reported that when co‐existing with nocturnal predators, the herbivores are more likely to have a diurnal activity pattern. In research done in Colombia, researchers reported that species adapt their behavior by increasing nocturnality to avoid human presence (Pardo et al. 2021). Wildlife diel activity patterns are generally shaped by evolutionary history but are flexibly adjusted in response to environmental stressors such as predation risk and human disturbance. These studies across various ecosystems have shown that species may adapt and shift their activity patterns to balance trade‐offs between safety, foraging efficiency, and environmental conditions.

Interestingly, for sambar deer, which exhibited similar activity patterns during the afternoon with the muntjacs, the activity at PTNP appears to be similar to other parts of Borneo in Kalimantan, but different from the observations by Tan et al. (2018), who reported sambar deer activity peaks during noon. This is particularly interesting as the sambar deer detection was 15, compared to 126 in PTNP. Based on research by Iannino et al. (2024), who compared the activity patterns obtained from GPS collars equipped with activity sensors and the camera trap images, they recommended that at least 75 detections are needed for a comparable diel activity pattern estimation.

Camera trapping has been increasingly used in determining the occupancy and abundance of species (e.g., Shannon et al. 2014; Burton et al. 2015). However, when studying elusive species with lower detections, it may produce less accurate estimations and therefore needs to be interpreted with caution. Regardless, the “golden standard” of diel activity pattern estimation using GPS collars with accelerometers can be financially burdensome for research projects and more invasive to the species studied. The advancement of technologies and applications to estimate diel activity patterns of wildlife using camera trap data have allowed for a non‐invasive approach to studying rare, elusive, and nocturnal species (Sollmann et al. 2013). However, it is important to note that the use of camera trap data needs to consider several disadvantages and biases, such as the placement of cameras, limited range of camera detection, and the fact that the cameras only record a single point in space and time per photograph, which can poorly represent the heterogeneous space‐use by wildlife, all of which potentially incorporate bias when interpreting the activity patterns (Burton et al. 2015; Kays et al. 2021; Tanwar et al. 2021).

This study presents a list of mammal species detected in a high‐altitude protected area, highlighting their richness and wide distribution in these forests. The findings underscore the ecological importance of high‐altitude habitats as potential refugia for biodiversity in the face of climate change. Furthermore, the spatio‐temporal patterns observed contribute valuable insights toward understanding species interactions within high‐altitude ecosystems, helping to fill existing knowledge gaps. We hope this study can help inform future species conservation and management strategies. Nonetheless, further research that covers areas we failed to reach in this study is essential to better understand the full picture of ecological dynamics and conservation needs of these understudied environments.

## Author Contributions

**Mufeng Voon:** conceptualization (lead), data curation (lead), formal analysis (lead), funding acquisition (lead), investigation (lead), methodology (lead), project administration (lead), resources (lead), software (lead), supervision (lead), validation (lead), visualization (lead), writing – original draft (lead), writing – review and editing (lead). **Ai Suzuki:** conceptualization (equal), data curation (equal), formal analysis (supporting), funding acquisition (supporting), investigation (equal), methodology (equal), project administration (supporting), resources (supporting), supervision (supporting), validation (equal), visualization (supporting), writing – review and editing (equal). **Shinya Numata:** conceptualization (supporting), data curation (supporting), formal analysis (supporting), funding acquisition (supporting), methodology (supporting), resources (supporting), software (supporting), supervision (supporting), validation (supporting), writing – review and editing (supporting). **Takafumi Mizuno:** data curation (supporting), formal analysis (equal), funding acquisition (supporting), investigation (supporting), project administration (supporting), resources (supporting), supervision (supporting), validation (supporting), writing – review and editing (supporting). **Melvin Gumal:** conceptualization (supporting), data curation (supporting), funding acquisition (supporting), project administration (supporting), resources (supporting), validation (equal), writing – review and editing (supporting).

## Conflicts of Interest

The authors declare no conflicts of interest.

## Supporting information


**Data S1:** ece371915‐sup‐0001‐DataS1.xlsx.

**Data S2:** ece371915‐sup‐0002‐DataS2.csv.

**Table S1:** ece371915‐sup‐0003‐TableS1.pdf.

## Data Availability

All the required data are uploaded as Supporting Information.
