# Supplementary material for: Non‐Volant Mammalian Diversity, Occurrence, and Ecological Patterns in a Tropical Montane Forest in Sarawak, Borneo
Source: Ecol Evol. 2025 Aug 12;15(8):e71915. doi: 10.1002/ece3.71915 (PMC12343747; doi:10.1002/ece3.71915)
Supplement: Supplementary file 3 — Table S1: ece371915‐sup‐0003‐TableS1.pdf. [file ECE3-15-e71915-s001.pdf]

Species	Banded civet	Bay cat	Bearded pig	Binturong	Bornean porcupine
Banded civet	NA	1	1	1	0.334101926
Bay cat	NA	NA	1	1	1
Bearded pig	NA	NA	NA	1	1
Binturong	NA	NA	NA	NA	1
Bornean porcupine	NA	NA	NA	NA	NA
Bornean yellow muntjac	NA	NA	NA	NA	NA
Collared mongoose	NA	NA	NA	NA	NA
Common palm civet	NA	NA	NA	NA	NA
Hose's civet	NA	NA	NA	NA	NA
Hose's langur	NA	NA	NA	NA	NA
Linsang	NA	NA	NA	NA	NA
Long-tailed macaque	NA	NA	NA	NA	NA
Long-tailed porcupine	NA	NA	NA	NA	NA
Malay civet	NA	NA	NA	NA	NA
Malay weasel	NA	NA	NA	NA	NA
Malayan porcupine	NA	NA	NA	NA	NA
Marbled cat	NA	NA	NA	NA	NA
Masked palm civet	NA	NA	NA	NA	NA
Moonrat	NA	NA	NA	NA	NA
Mousedeer spp.	NA	NA	NA	NA	NA
North Borneo gibbon	NA	NA	NA	NA	NA
Otter civet	NA	NA	NA	NA	NA
Pig-tailed macaque	NA	NA	NA	NA	NA
Red langur	NA	NA	NA	NA	NA
Red muntjac	NA	NA	NA	NA	NA
Sambar deer	NA	NA	NA	NA	NA
Short-tailed mongoose	NA	NA	NA	NA	NA
Small-clawed otter	NA	NA	NA	NA	NA
Sun bear	NA	NA	NA	NA	NA
Sunda clouded leopard	NA	NA	NA	NA	NA
Sunda leopard cat	NA	NA	NA	NA	NA
Sunda pangolin	NA	NA	NA	NA	NA
Tufted ground squirrel	NA	NA	NA	NA	NA
Yellow-throated marten	NA	NA	NA	NA	NA

Bornean yellow muntjac	Collared mongoose	Common palm civet	Hose's civet	Hose's langur
7.78E-22	0.401879597	1	6.00E-06	1
0.198524325	1	1	1	1
0.161724566	1	1	1	1
0.000188873	1	1	1	1
6.36E-05	0.000330986	1	5.24E-12	1
NA	1.38E-14	0.436959666	5.78E-44	0.3413727
NA	NA	1	1	1
NA	NA	NA	1	1
NA	NA	NA	NA	1
NA	NA	NA	NA	NA
NA	NA	NA	NA	NA
NA	NA	NA	NA	NA
NA	NA	NA	NA	NA
NA	NA	NA	NA	NA
NA	NA	NA	NA	NA
NA	NA	NA	NA	NA
NA	NA	NA	NA	NA
NA	NA	NA	NA	NA
NA	NA	NA	NA	NA
NA	NA	NA	NA	NA
NA	NA	NA	NA	NA
NA	NA	NA	NA	NA
NA	NA	NA	NA	NA
NA	NA	NA	NA	NA
NA	NA	NA	NA	NA
NA	NA	NA	NA	NA
NA	NA	NA	NA	NA
NA	NA	NA	NA	NA
NA	NA	NA	NA	NA
NA	NA	NA	NA	NA
NA	NA	NA	NA	NA
NA	NA	NA	NA	NA
NA	NA	NA	NA	NA
NA	NA	NA	NA	NA

Linsang	Long-tailed macaque	Long-tailed porcupine	Malay civet	Malay weasel
0.357025631	4.83E-09	1	1.01E-12	1
1	0.435453834	1	0.316029793	1
1	0.653021543	1	0.37526111	1
1	0.003647203	1	0.00112176	1
0.000131626	0.1096466	0.312947047	0.010202063	1
6.66E-17	1	9.55E-21	1	0.011972994
1	2.57E-10	0.568006698	5.96E-12	1
1	1	1	0.884833569	1
1	2.42E-22	3.85E-05	4.64E-29	1
1	1	1	0.653812073	1
NA	2.27E-11	0.536798578	1.99E-13	1
NA	NA	6.24E-09	1	0.041996195
NA	NA	NA	2.04E-12	1
NA	NA	NA	NA	0.024918306
NA	NA	NA	NA	NA
NA	NA	NA	NA	NA
NA	NA	NA	NA	NA
NA	NA	NA	NA	NA
NA	NA	NA	NA	NA
NA	NA	NA	NA	NA
NA	NA	NA	NA	NA
NA	NA	NA	NA	NA
NA	NA	NA	NA	NA
NA	NA	NA	NA	NA
NA	NA	NA	NA	NA
NA	NA	NA	NA	NA
NA	NA	NA	NA	NA
NA	NA	NA	NA	NA
NA	NA	NA	NA	NA
NA	NA	NA	NA	NA
NA	NA	NA	NA	NA
NA	NA	NA	NA	NA
NA	NA	NA	NA	NA

Malayan porcupine	Marbled cat	Masked palm civet	Moonrat	Mousedeer spp.
1	4.15E-08	9.01E-07	0.000110321	2.46E-29
1	1	1	1	0.225549685
1	0.584176862	1	1	0.184585243
1	1	1	1	0.000185445
1	2.95E-13	3.42E-12	1.51E-09	9.53E-06
1.76E-15	3.69E-32	2.73E-34	3.83E-28	1
0.025653032	1	1	1	2.58E-15
1	0.503473877	1	1	0.502833553
1.59E-09	1	1	1	2.44E-55
1	1	1	1	0.390040731
0.015564117	1	1	1	4.33E-18
4.11E-06	3.88E-22	1.46E-21	3.47E-18	1
1	1.32E-07	3.26E-06	0.000280685	4.30E-27
7.84E-09	7.49E-26	4.72E-26	9.05E-22	1
1	1	1	1	0.013694778
NA	1.27E-10	1.59E-09	5.84E-07	1.77E-20
NA	NA	1	1	6.49E-35
NA	NA	NA	1	1.45E-38
NA	NA	NA	NA	4.19E-31
NA	NA	NA	NA	NA
NA	NA	NA	NA	NA
NA	NA	NA	NA	NA
NA	NA	NA	NA	NA
NA	NA	NA	NA	NA
NA	NA	NA	NA	NA
NA	NA	NA	NA	NA
NA	NA	NA	NA	NA
NA	NA	NA	NA	NA
NA	NA	NA	NA	NA
NA	NA	NA	NA	NA
NA	NA	NA	NA	NA
NA	NA	NA	NA	NA
NA	NA	NA	NA	NA
NA	NA	NA	NA	NA

North Borneo gibbon	Otter civet	Pig-tailed macaque	Red langur	Red muntjac	Sambar deer
1	1	1	1	0.026306215	5.71E-06
1	1	1	1	1	1
1	1	1	1	1	1
1	1	1	1	1	0.193697779
1	1	1	1	1.61E-08	1
1	1	2.01E-18	0.012856983	5.11E-52	0.513038689
1	0.629695764	0.004030939	1	1	1.78E-07
1	1	1	1	1	1
1	0.58296435	8.03E-15	1	0.217932284	1.38E-20
1	1	1	1	1	1
1	0.758144365	0.001572596	1	1	2.20E-08
1	1	3.64E-06	0.035484509	3.78E-19	1
1	1	1	1	0.161064401	7.67E-06
1	1	2.25E-09	0.023242329	1.84E-27	1
1	1	1	1	1	0.518864141
1	1	1	1	2.67E-06	0.005010081
1	0.074014996	5.45E-13	1	0.000210003	3.58E-19
1	0.204574399	2.04E-12	1	0.006869383	8.50E-19
1	0.295501293	4.48E-09	1	0.188457084	3.54E-15
1	1	7.61E-28	0.014709597	7.39E-85	0.349076046
NA	1	1	1	1	1
NA	NA	1	0.713189964	1	1
NA	NA	NA	1	9.03E-17	0.00486727
NA	NA	NA	NA	1	0.333972453
NA	NA	NA	NA	NA	2.40E-18
NA	NA	NA	NA	NA	NA
NA	NA	NA	NA	NA	NA
NA	NA	NA	NA	NA	NA
NA	NA	NA	NA	NA	NA
NA	NA	NA	NA	NA	NA
NA	NA	NA	NA	NA	NA
NA	NA	NA	NA	NA	NA
NA	NA	NA	NA	NA	NA
NA	NA	NA	NA	NA	NA

Short-tailed mongoose	Small-clawed otter	Sun bear	Sunda clouded leopard	Sunda leopard cat
1	1	1	1	1
1	1	1	1	1
1	1	1	1	1
1	1	1	1	1
0.010871781	1	0.000211463	0.007241217	0.721308159
1.01E-19	1	3.01E-22	4.87E-12	7.77E-10
1	1	1	1	1
1	1	1	1	1
0.790230448	1	1	1	1
1	1	1	1	1
1	1	1	1	1
2.48E-10	1	1.91E-12	1.99E-08	4.44E-06
1	1	1	1	1
2.07E-13	1	9.45E-16	8.24E-10	1.85E-07
1	1	1	1	1
1	1	0.050633048	0.337350692	1
0.000680959	1	0.042328671	1	0.011992038
0.020769526	1	0.885350482	1	0.232442854
0.232418016	1	1	1	1
2.90E-23	1	1.02E-25	1.39E-12	1.64E-10
0.993644076	1	1	1	1
1	1	1	1	1
0.115283994	1	0.001575979	0.079596814	1
1	1	1	1	1
1	1	1	1	1
3.08E-07	1	2.24E-09	9.86E-06	0.00204601
NA	1	1	1	1
NA	NA	1	1	1
NA	NA	NA	1	1
NA	NA	NA	NA	1
NA	NA	NA	NA	NA
NA	NA	NA	NA	NA
NA	NA	NA	NA	NA
NA	NA	NA	NA	NA

Sunda pangolin	Tufted ground squirrel	Yellow-throated marten
1	1	1.86E-07
1	1	1
1	1	1
1	1	1
1	1	9.85E-14
0.009658312	2.00E-10	4.50E-50
1	0.622487754	1
1	1	1
1	0.002506016	1
1	1	1
1	0.6573	1
0.038337	2.34E-05	2.20E-24
1	1	2.00E-06
0.021678382	4.47E-07	3.98E-32
1	1	1
1	1	1.45E-11
1	2.04E-06	1
1	6.56E-05	1
1	0.001660305	1
0.010984653	8.21E-12	3.89E-65
1	1	1
1	1	0.531130604
1	1	1.45E-18
1	1	1
1	1	0.023406467
0.544028599	0.015097009	2.80E-23
1	1	0.315362543
1	1	1
1	1	1
1	1	1
1	1	1
NA	1	1
NA	NA	0.000570601
NA	NA	NA
